# Role of ^18^F-FDG PET/CT in patients without known primary malignancy with skeletal lesions suspicious for cancer metastasis

**DOI:** 10.1371/journal.pone.0196808

**Published:** 2018-05-10

**Authors:** Soo Bin Park, Jung Mi Park, Seung Hwan Moon, Young Seok Cho, Jong-Mu Sun, Byung-Tae Kim, Kyung-Han Lee

**Affiliations:** 1 Department of Radiology, Soonchunhyang University Seoul Hospital, Seoul, Korea; 2 Department of Nuclear Medicine, Soonchunhyang University Bucheon Hospital, Gyeonggi, Korea; 3 Department of Nuclear Medicine, Samsung Medical Center, Sungkyunkwan University School of Medicine, Seoul, Korea; 4 Division of Hematology-Oncology, Department of Medicine, Samsung Medical Center, Sungkyunkwan University School of Medicine, Seoul, Korea; GERMANY

## Abstract

**Background:**

When subjects without a known malignancy present with suspicious skeletal lesions, differential diagnosis and primary cancer identification is important. Here, we investigated the role of FDG PET/CT in this clinical situation.

**Methods:**

We enrolled 103 patients with no known malignancies who were referred for FDG PET/CT because of bone lesions that were suspicious for cancer metastasis. Each extra-skeletal FDG lesion was categorized as consistent with primary cancer or with metastasis based on the distribution and pattern of all abnormal lesions in the individual.

**Results:**

Final diagnosis revealed that bone lesions represented cancer metastasis in 75 patients (72.8%). In the remaining 28 patients (27.2%), they were from other causes including multiple myeloma or lymphoma, malignant primary bone tumor, and benign bone disease. PET/CT indicated a primary cancer in 70 patients (68.0%). This was the correct primary site in 46 cases and the incorrect site in 13 cases (including 6 cases with cancer of unknown primary, CUP). In the remaining 11 cases, the bone lesions were due to other causes. PET/CT did not indicate a primary cancer in 33 patients (32.0%). Of these cases, 17 did not have a primary cancer, 8 had CUP, and 8 had primary cancers that were missed. Thus, PET/CT had a sensitivity of 61.3% and specificity of 60.7% for primary cancer identification in the entire population. Excluding patients with CUP, PET/CT sensitivity was 75.4%. PET/CT also provided information useful for recognizing multiple myeloma and benign bone disease as the cause of the skeletal lesions.

**Conclusions:**

In patients without known malignancies with suspected skeletal cancer metastasis, FDG PET/CT can help identify the primary cancer and provide useful information for differential diagnosis.

## Introduction

The bone is the most common organ for spread of malignant tumors [1,2]. Furthermore, it is not uncommon for subjects to present with metastatic bone lesions as the first manifestation of cancer disease [3–6]. Because metastatic bone disease frequently remains confined, patient outcome is highly dependent on prevention and treatment of skeletal complications [1,2]. Therefore, when individuals not recognized to have malignant disease demonstrate suspicious skeletal lesions, it is important to promptly determine the underlying cause. If cancer metastasis is responsible, it is further necessary to identify the primary malignancy for appropriate treatment planning. However, the search for primary cancer through multiple imaging studies can be time-consuming and biopsies are invasive. Hence, these patients would benefit from a more efficient method for differential diagnosis and primary cancer identification.

^18^F-fluorodeoxyglucose positron emission tomography/CT (FDG PET/CT) has a well-established role in cancer diagnosis, staging and treatment response monitoring [7,8]. FDG PET/CT is also useful for differential diagnosis of suspected bone lesions in patients with known cancer [9,10]. When patients who do not have diagnosed cancer disease initially present with suspicious bone lesions, a completely different investigative challenge arises. In this situation, the ability of PET/CT to screen the entire body in a highly sensitive manner provides an opportunity to search for potential primary malignancies. The few previous studies that explored this issue included only small numbers of patients with established skeletal cancer metastasis [11,12].

In this study, we investigated the role of FDG PET/CT for differential diagnosis and primary cancer identification in patients without a known primary malignancy who were referred for clinically suspected but unconfirmed skeletal metastasis.

## Materials and methods

### Subjects

Study subjects were 103 patients with no known malignancies at presentation who underwent FDG PET/CT at our institution between 2003 and 2013 for abnormal bone lesions radiologically suspected as cancer metastasis. Patients with known malignancy were excluded, and there were no other exclusion criteria. Radiologic suspicion of bone metastases was based on CT findings in 94, MR in 7, and bone scan in 2 patients. CT findings suspicious for bone metastasis were multiple osteolytic or osteosclerotic lesions (n = 83), single osteolytic bone lesion (n = 7), and suspected pathologic fractures (n = 4). For MR, it was space occupying bone lesions with contrast enhancement. For bone scan, it was multiple skeletal lesions with increased uptake.

Among our study subjects, 94.2% (n = 97) underwent one or more radiologic study other than PET/CT. This includes chest CT in 88, abdomen and pelvis CT in 82, bone scan in 45, abdomen US or MRI in 10, neck US or CT or MRI in 15, mammogram or breast US or MRI in 28, and prostate US in 6 cases. These radiologic studies offered findings suggesting a primary malignancy in 46 cases, which turned out to be correct in 38 cases (S1 Table). In addition, gastroduodenoscopy or colonoscopy were performed after PET/CT studies in 21 and 27 patients, respectively (4 patients had both). This led to the diagnosis of 4 gastric cancers and 3 colon cancers.

All analyses were in accordance with the ethical standards of the institutional research committee and with the principles of the 1964 Declaration of Helsinki and its later amendments or comparable ethical standards. Our institutional review board approved this retrospective study and the requirement for written consent was waived.

### FDG PET/CT acquisition

PET/CT was performed on a Discovery LS or Discovery STe scanner (GE Healthcare, Milwaukee, WI). CT was performed 60 min after injection of 5.5 MBq/kg FDG without intravenous or oral contrast and with free-breathing, following a protocol for anatomical localization of the PET images and attenuation correction. Continuous spiral acquisition was performed on an 8-slice helical CT with 140 KV and 40–120 mA adjusted to body weight (Discovery LS; section width, 5 mm), or a 16-slice helical CT with 140 KV and 30–170 mA on auto-mA mode (Discovery STe; section width, 3.75 mm). Emission scans were then obtained at 4 min per frame in 2D mode and 2.5 min per frame in 3D mode, respectively. Attenuation-corrected PET images with voxel size of 4.3 × 4.3 × 3.9 mm and 3.9 × 3.9 × 3.3 mm were reconstructed, respectively, by 2D and 3D ordered-subset expectation maximization algorithms (28 subsets and 20 subsets, respectively; 2 iterations).

### Analysis of PET/CT images

Two nuclear medicine physicians blinded to the final diagnosis reviewed attenuation-corrected PET, CT and fused PET/CT images in axial, coronal and sagittal planes on a workstation. FDG PET images were reviewed to identify all skeletal and extra-skeletal lesions. Given that our CT acquisition protocol could limit the detection of small lesions, we payed special attention to identify potential extra-skeletal lesions of small size or low FDG uptake, including those in the lung.

Because CT images were not contrast-enhanced, FDG positive lesions with no correlating CT lesion were also considered positive. CT positive lesions with mild FDG uptake were considered positive if they were consistent with cancers with low FDG avidity such as lung adenocarcinoma, hepatobiliary cancer, and renal cell carcinoma. However, CT positive lesions with no FDG uptake were considered benign lesions and negative for malignancy. In each individual patient, the extra-skeletal FDG lesion that was most likely to spread to the remaining abnormal lesions were taken to indicate the primary cancer. If no such site was identified, PET/CT was considered negative for primary cancer. Extra-skeletal FDG accumulations attributed to physiologic activity, inflammatory disease or benign tumors were considered insignificant.

### Final diagnosis

Final diagnosis was by biopsy of bone lesions (n = 68) or extra-skeletal lesions (n = 45) in conjunction with comprehensive assessment of multiple imaging studies. Diagnosis other than solid cancer metastasis included skeletal involvement of hematologic malignancies (multiple myeloma or lymphoma), malignant primary bone tumors, and benign bone diseases. In patients diagnosed with solid cancer metastasis to the bone, the primary cancer was finally determined by tissue biopsy or comprehensive analysis of clinical, laboratory, imaging, and endoscopy results.

### Statistical analysis

Differences in variables between groups were compared by McNemar’s tests or chi square tests. PET/CT performance for identification of primary malignancy was subject-based. Statistical analyses were performed using PASW Statistics-18 (IBM Corporation, Somers, NY).

## Results

### Clinical characteristics and FDG PET/CT findings of the skeletal lesions

The 103 study subjects had a mean age of 60 ± 13 y (range, 14 to 88 y), and 66% were males. PET demonstrated a single skeletal FDG lesion in 16, 2–4 lesions in 14, and ≥5 lesions in 72 patients. One subject showed no FDG bone lesion despite multiple bone lesions on MRI. The FDG lesions were on axial bone only in 27, peripheral bone only in 1, and both axial and peripheral bone in 74 subjects.

### Final diagnosis of study subjects

Final diagnosis revealed that the bone lesions represented solid cancer metastasis in 75 patients (72.8%), whereas they were due to other causes in 28 patients (27.2%). Among the former, the primary cancer was finally identified in 61 subjects, while it remained undetermined as cancer of unknown primary (CUP) in 14 subjects. Since 2 patients had double primaries, a total of 63 cancers were identified (Table 1). The primary malignancy was confirmed by biopsy in 42 cases. Of these, biopsy was from the primary tumor in 33 cases. This included 11 biopsies during gastrointestinal endoscopy (performed in 44 patients), 6 prostate biopsies, 4 breast biopsies, 4 lung biopsies, 2 thyroid biopsies, and 2 kidney biopsies. In the remaining 9 cases, biopsy was from metastatic lesions. The most frequent primary was lung cancer (39.7%), followed by gastric, prostate, kidney and hepatobiliary cancers.

**Table 1:** PET/CT interpretation of a total of 63 primary cancers from 61 subjects.

Primary cancer	Number of cancers	Final diagnosis		FDG PET/CT	
		Biopsy	Clinical	Identified	Missed
Lung cancer	25	12	13	20	5
Gastric cancer	6	5	1	4	2
Prostate cancer	6	6	0	4^a^^,^^b^	2
Hepatobiliary cancer	6	2	4	5	1
Kidney cancer	5	4	1	3	2^c^
Colorectal cancer	4	3	1	4^b^	0
Breast cancer	4	4	0	3	1
Thyroid cancer	2	2	0	2	0
Pancreas cancer	1	0	1	1	0
Esophagus cancer	1	1	0	1^c^	0
Malignant melanoma Sarcoma	1 2	1 2	0 0	0 1	1 1
Total number	63	42	21	48	15

a, one subject had synchronous multiple myeloma

b, one subject had synchronous rectal and prostate cancer (both detected by PET/CT)

c, one subject had synchronous esophageal (detected by PET/CT) and renal cell cancer (missed by PET/CT).

The 28 patients with other causes for the bone lesions included 12 with hematologic malignancies (8 multiple myelomas and 4 lymphomas), 4 with primary bone malignancy, and 12 with benign bone disease. The latter included bone trauma, benign bone tumor, SAPHO syndrome and degenerative disease.

### PET/CT positive for primary cancer

**Fig 1:**
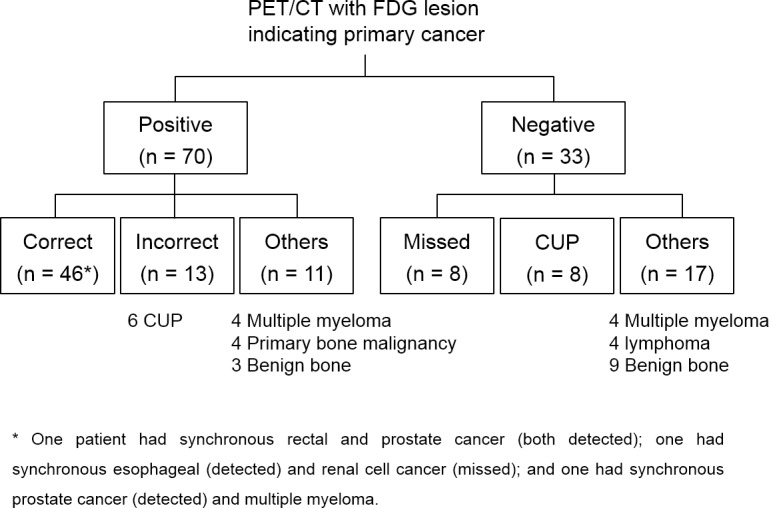
Final diagnosis of subjects with positive and negative PET/CT results for indicating the primary cancer.

The final diagnosis of subjects with positive and negative PET/CT results indicating the primary cancer is summarized in Fig 1. PET/CT indicated a primary cancer in 70 subjects (68.0%), and this proved to be correct in 46 cases (67.1%). Thus PET/CT correctly identified a total of 48 primary cancers from 46 patients among a total of 63 primary cancers finally diagnosed from 61 patients (Table 1). This included 20/25 confirmed lung cancers, 4/6 gastric cancers, 4/6 prostate cancers and 5/6 hepatobiliary cancers. It should be mentioned that PET/CT correctly identified all 13 primary cancers that were near the bladder or had low FDG uptake. This included 5 lung cancers, 1 hepatocellular carcinoma, 1 renal cell carcinoma, 4 prostate cancers, and 2 rectal cancers. In addition, all 3 gastric cancers and 4 colon cancers later diagnosed by gastrointestinal endoscopy were correctly identified by PET/CT.

In 13 cases (18.6%), the site indicated by PET/CT was incorrect: 7 cases had a primary cancer at a different site and 6 cases had CUP. In the 11 cases remaining (15.7%), the bone lesions were due to other causes including multiple myeloma (n = 4), malignant primary bone tumor (n = 4), and benign bone disease (n = 3; Fig 1).

Several patients showed additional extra-skeletal FDG lesions that appeared more consistent with metastatic disease. This included FDG uptake in lymph nodes (n = 45), lung (n = 16), liver (n = 7), pleura (n = 6), adrenal (n = 6), and other sites (n = 9).

### PET/CT negative for primary cancer

PET/CT was unable to indicate a primary cancer in 33 subjects (32.0%; Fig 1; Table 2). In 17 of these cases (42.4%), the bone lesions were caused not by solid cancer metastasis but by involvement of multiple myeloma (n = 4) or lymphoma (n = 4), or by benign bone diseases (n = 9; Fig 1). In 8 cases (24.2%), PET/CT missed a primary cancer that was finally diagnosed. In another 8 cases, bone metastasis was present but the primary cancer remained undetermined (CUP). In this group, 12 cases showed FDG uptake in lymph nodes that were considered consistent with metastatic disease.

**Table 2:** Final diagnosis of patients that were PET/CT negative for primary cancer.

Final diagnosis	Number of subjects	No extra-skeletal FDG lesion	Lymph node FDG lesion only
Lung cancer	2	0	2
Prostate cancer	2	2	0
Sarcoma	1	1	0
Gastric cancer	1	1	0
Kidney cancer	1	0	1
Cholangiocarcinoma	1	1	0
CUP	8	1	6
Lymphoma	4	1	3
Multiple myeloma	4	4	0
Benign bone disease	9	9	0
Total	33	20	12

CUP, carcinoma of unknown primary

### PET/CT performance for primary cancer identification and differential diagnosis

**Fig 2:**
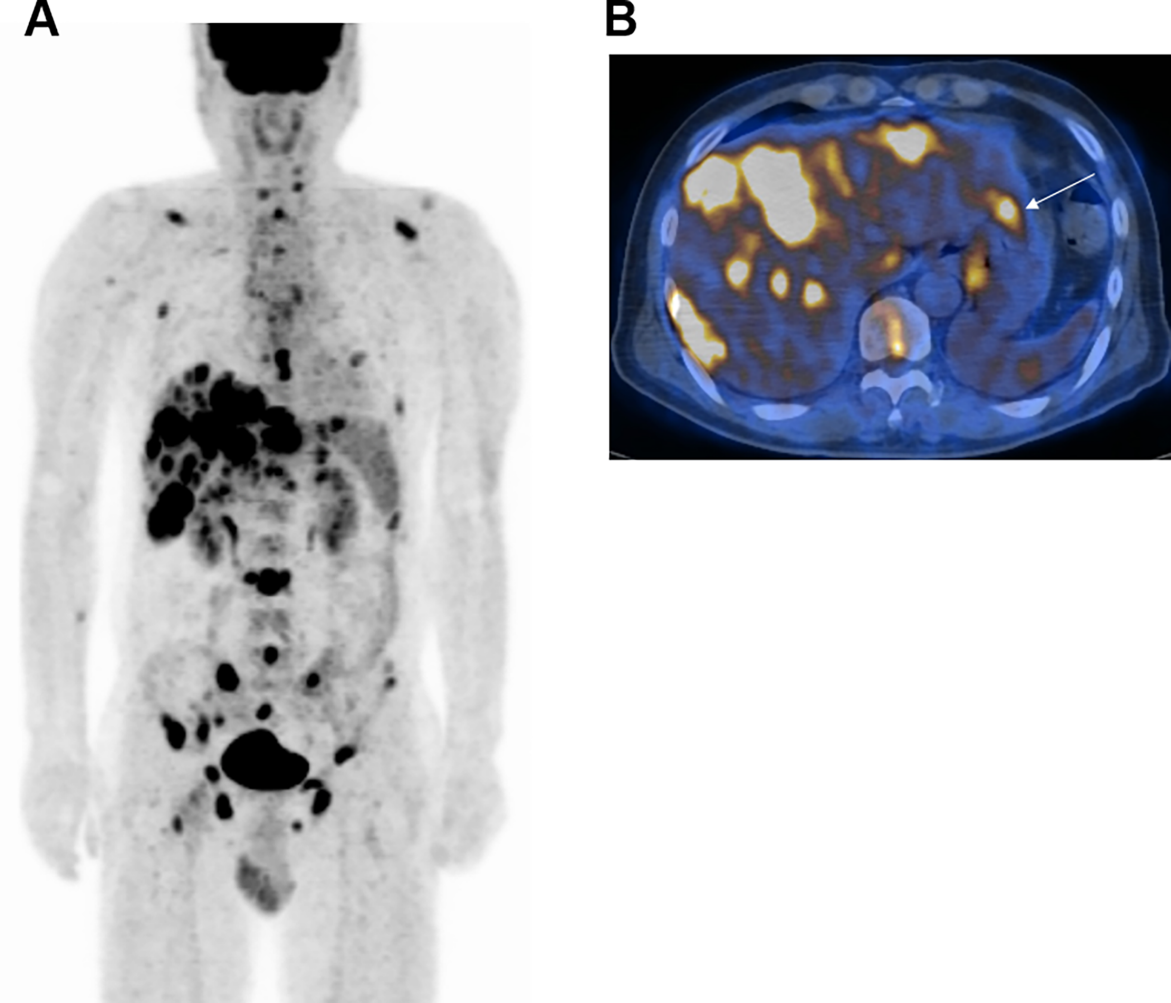
A 55-year-old male with gastric cancer identified by FDG PET/CT. (a) The maximum intensity projection image shows multiple hypermetabolic metastatic lesions in the skeleton, liver and hepatoduodenal lymph nodes. (b) A FDG lesion was detected in the stomach that was interpreted as primary gastric cancer (arrow). Endoscopy revealed a 3-cm-sized ulcerative gastric mass and biopsy confirmed adenocarcinoma of the stomach.

**Fig 3:**
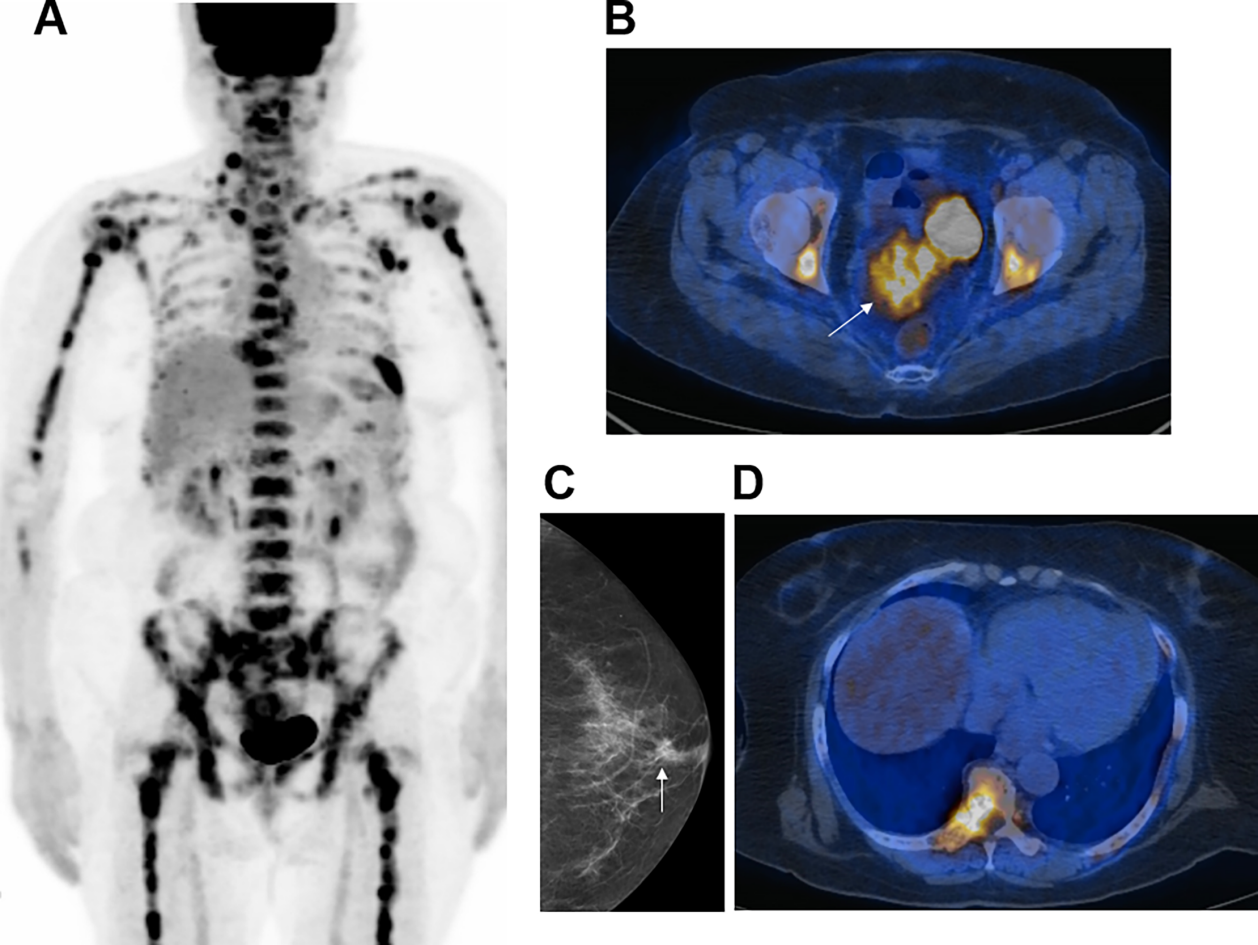
A 65-year-old female with breast cancer missed by FDG PET/CT. (a) The maximum intensity projection image shows multiple hypermetabolic lesions in the skeleton and in the axillary, supraclavicular, cervical and mediastinal lymph nodes. (b) A FDG lesion was detected in the uterus that was interpreted as the primary malignancy (arrow). However, endometrial curettage biopsy confirmed metastasis from poorly differentiated carcinoma. (c) Mammography revealed a 0.9 cm subareolar nodule (arrow) (d) that was FDG non-avid on PET/CT. Biopsy confirmed invasive breast carcinoma.

Among the entire study population, PET/CT identified the primary cancer with a sensitivity of 61.3% (46/75), specificity of 60.7% (17/28) and accuracy of 61.2% (63/103). Excluding patients with CUP, PET/CT could detect 75.4% (46/61) of all primary cancers that were finally diagnosed. Representative cases in which FDG PET/CT correctly identified and missed the primary cancer are illustrated in Figs 2 and 3, respectively.

As a PET/CT finding that might help identify patients with lung cancer, increased FDG uptake in mediastinal lymph nodes occurred significantly more often compared to those with other primary cancers (68.0 vs. 16.7%, *P* <0.001).

PET/CT also provided information helpful for differential diagnosis. Hence, patients with multiple myeloma displayed osteolytic bone lesions (7/9) and absence of extra-skeletal FDG lesions (7/9) significantly more frequently than those with other conditions (3/94 and 17/94, respectively; Table 3). This was also true among patients with PET/CT that was negative for primary cancer (5/5 vs. 17/29 and 5/5 vs. 3/29, respectively). In addition, skeletal lesions from benign bone disease were frequently FDG non-avid (n = 5/12) and often displayed benign CT features consistent with bone trauma, SAPHO syndrome or degenerative disease (n = 4/12).

**Table 3:** PET/CT features indicating multiple myeloma as cause for bone lesions.

	Multiple myeloma	Others	*P* value
**All patients**	(n = 9)	(n = 94)	
No extra-skeletal FDG lesion	7	17	< 0.001
Osteolytic CT change	7	3	< 0.001
Both	6	3	< 0.001
**Patients PET/CT negative** **for primary cancer**	(n = 5)	(n = 29)	
No extra-skeletal FDG lesion	5	17	0.074
Osteolytic CT change	5	3	< 0.001
Both	5	3	< 0.001

## Discussion

The subjects of this study were individuals with no known malignancies who were referred because of skeletal lesions that were suspicious for cancer metastasis. Our results demonstrated that FDG PET/CT has a useful role in this clinical setting by screening the whole body for potential primary cancers as well as by providing evidence that can identify alternative causes for the bone lesions.

The suspected bone lesions were revealed to actually represent metastasis from a solid cancer in 72.8% of our patients. Among these subjects, the primary malignancy was finally identified in 81.3% of cases. This is comparable to the rate of primary cancer diagnosis in patients presenting with skeletal metastasis of unknown origin [13]. Cancers arising from the lung, breast and prostate have a propensity to spread to the bone [1]. In our study, lung cancer was by far the most frequent origin of the metastatic bone lesions, comprising one third of identified primary cancers. This high occurrence of lung cancer as the primary is consistent with previous observations in patients with bone and soft-tissue metastasis [11–13]. In our study, prostate, gastric and hepatobiliary cancers were next in frequency, followed by kidney, breast and colorectal cancer. Shimada et al. showed that the primary cancers responsible for bone metastasis differ between patients with known and unknown origins at presentation [11]. Their study showed that the most frequent primary in the former group was breast cancer. In contrast, the primary in the latter group was most frequently lung cancer, followed by prostate, kidney, gastric and colorectal cancer [11]. This distribution of frequent primary cancers is similar to our findings.

In this study, we addressed two different issues; namely, the differentiation of the cause for the suspected bone lesions and the identification of primary tumor sites. As for the first issue, in addition to the 75 cases with actual cancer metastasis, our subjects also included 12 hematologic malignancies, 4 primary bone malignancies and 12 benign bone diseases. These lesions can all simulate metastasis on radiologic exams [14], but PET/CT might be usefulness for differential diagnosis [9, 15, 16]. In our study, patients with causes other than cancer metastasis generally did not show extra-skeletal FDG lesions. Furthermore, benign bone lesions were frequently FDG non-avid, in contrast to malignant bone lesions that all had moderate (> bone marrow uptake) or intense (SUVmax > 5.0) FDG uptake, and often displayed CT features consistent with bone trauma, SAPHO syndrome or degenerative disease.

As for the second issue, our results showed that FDG PET/CT identified the primary cancer with a sensitivity of 61.3%. As the most frequent primary, lung cancer was detected with a high 80.0% sensitivity. In the study by Shimada et al., FDG PET/CT detected the primary cancer in 43.6% (17/39) of patients with bone metastasis [11]. In another study, FDG PET/CT identified the primary in 50% (12/24) of patients with bone and soft-tissue metastasis from an unknown primary [12]. Hence, our sensitivity of FDG PET/CT for primary cancer identification appears slightly better compared to these previous studies that included smaller numbers of patients. A recent meta-analysis reported that PET/CT had a median detection rate of 36% for primary tumors in patients with extracervical metastases from cancers of unknown primary [17]. In our subjects with suspected skeletal metastases, the detection rate was 44.7% (46/103), which is comparable with that report, although the study population differs.

There is a relative paucity on studies exploring the role of FDG PET/CT for aiding differential diagnosis in subjects suspected of skeletal metastasis. With more than a quarter of study subjects having bone lesions from causes other than cancer metastasis, we could obtain a 60.7% diagnostic specificity for primary cancer identification. Additionally, we found that PET/CT might provide information useful for distinguishing underlying conditions. For example, osteolytic bone change and absence of extra-skeletal FDG lesions appeared useful for recognizing patients with multiple myeloma, where skeletal FDG uptake represents bone marrow involvement by malignant plasma cells [18, 19]. However, it should be noted that the number of cases with primary bone tumor, multiple myeloma and bone lymphoma in our study was small. While this implies a low incidence of these entities among patients with suspicious bone metastasis, it also warrants further studies to clarify the role of FDG PET/CT for identifying such causes.

Limitations of our study include its retrospective design. Also, including patients with bone lesions from other causes to patients with cancer bone metastasis deterred the homogeneity of our study population. However, this study was intentionally designed in this manner to simulate clinical situations where patients are referred for PET/CT due to suspicious bone lesions whose nature has not yet been established.

In conclusion, in patients with no known malignancies who present with skeletal lesions clinically suspected of cancer metastasis, FDG PET/CT can identify potential primary cancers and can also aid in differential diagnosis by recognizing other causes for the bone lesions.

## Supporting information

S1 Table Primary malignancies suggested by non-PET/CT imaging studies. (DOCX)

S1 File (XLSX)

## Abbreviations

PET/CT: positron emission tomography/computed tomography
FDG: 2-deoxy-2-[18F]fluoro-D-glucose
SUVmax: maximum standardized uptake value
CUP: cancer of unknown primary
