# Supplementary material for: Role of 18F-FDG PET/CT in patients without known primary malignancy with skeletal lesions suspicious for cancer metastasis
Source: PLoS One. 2018 May 10;13(5):e0196808. doi: 10.1371/journal.pone.0196808 (PMC5945029; doi:10.1371/journal.pone.0196808)
Supplement: S1 Table — (DOCX) [file pone.0196808.s001.docx]

**Supporting information**

**S1 Table. Primary malignancies suggested by non-PET/CT imaging studies**

| Primary cancer | Number of cancers | Suggested by non-PET/CT imaging |
| --- | --- | --- |
| Lung cancer | 25 | 14 |
| Gastric cancer | 6 | 1 |
| Prostate cancer | 6 | 4 |
| Hepatobiliary cancer | 6 | 2 |
| Kidney cancer | 5 | 4 |
| Colorectal cancer | 4 | 4 |
| Breast cancer | 4 | 4 |
| Thyroid cancer | 2 | 2 |
| Pancreas cancer | 1 | 1 |
| Esophagus cancer | 1 | 1 |
| Malignant melanoma  Sarcoma | 1  2 | 0  1 |
| Total number | 63 | 38 |
